# Experiments with Recently Recommended Remedies in Gonorrhœa

**Published:** 1884-04

**Authors:** E. L. Keyes


### EXPERIMENTS WITH RECENTLY RECOMMENDED REMEDIES IN GONORRHŒA.

BY E. L. KEYES, M.D.

[Journal of Cutaneous and Venereal Diseases.]

Hot water has become one of the therapeutical modes of the day, and irrigation and drainage, as surgical principles, have been brought to bear upon a number of maladies formerly treated in other ways.

How long ago these methods were introduced into the management of gonorrhœal maladies I have not sought to find out, but doubtless investigation would prove them to be very ancient.

I remember that in 1865, in the Hotel Dieu, at Paris, Maissoneuve had an apparatus consisting of a suspended pail of water and a rubber tube working as a siphon with a special terminal nozzle capable of being made large near its proximal end, by which he attempted to cure specific vaginal inflammation in women by the principle of moderate distention of the vagina associated with incessant irrigation, night and day.

The method did not find favor or success, as I observed it.

Somewhat later I devised a retrograde urethral irrigator for the male urethra, but nothing came of it. Reginald Harrison, of Liverpool, two years ago published a method of prolonged deep urethral irrigations by mild astringents as a certain method of relief in gleety conditions of the urethra, a method which I have employed in several instances without success.

In any case of urethral inflammation in the male, hot water irrigation, intermittently applied, is a part of the treatment instituted by nature, for every act of urination performed by the patient washes out the urethra with a hot fluid, generally rendered bland and alkaline by the alkaline diuretics prescribed, yet as a curative means this natural irrigation has little value.

In these modern days of hot-water therapeutics, however, the tendency is for the surgeon to take the initiative. The hot-water cure of dyspeptic and intestinal derangements, headache, and constipation has a large corps of adherents among the laity as well as in the profession, while hot water douches internal and external have acquired a just popularity for the good they effect in various morbid conditions of the stomach, vagina, uterus, bladder, and rectum.

It is but natural, therefore, that hot water should find its way into urethral therapeutics, and the subject seems now to be receiving general attention in this city, notably since a paper was published upon the subject recently by Dr. Curtis, in the *Medical Record*, in which hot irrigation followed by an astringent application of tannin was advocated for the abortion of a commencing gonorrhœal attack.

Dr. Otis, in his latest publication, speaks kindly of the hot-water method without claiming any personal results from its application in his own practice.

Besides the use of hot water by deep irrigation, a claim is made for its efficacy when repeatedly used by the ordinary method of syringing as customarily practiced by the patient.

I have come into contact with a few cases in which both these methods have been used, and my impression of them based upon these few cases is that they are not only useless but dangerous in many instances, especially in fresh gonorrhœa in a virgin subject. In the case of old sinners, whose urethral canals have been toughened by several previous inflammatory attacks, they appear to be harmless, sometimes even efficient.

The first case I have to record as bearing upon the subject is that of a young gentleman with his first urethritis, apparently a mild attack, certainly not a specific gonorrhœa.

He was referred to me by his physician after a failure of the hot-water method, to which, however, a mild stimulant had been added.

The history is the following:

*Three weeks* after exposure, a very mild urethral inflammation manifested itself to the patient by a moderate discharge. For this he was advised, commencing at the thirty-sixth hour of the mild discharge, to remain at home and inject his urethra hourly with water as hot as he could comfortably tolerate, and twice a day to employ an injection containing one-quarter of a grain of nitrate of silver to the ounce.

The injections always caused pain. After the first injection a little blood began to flow and the urethral orifice to swell moderately. The process was continued until the tenth day, when the patient was sent to me with considerable redness and œdema of the prepuce (especially in the region of the frenum) and a swollen painful gland in the groin; great pain on making water, very moderate urethral flow, frequency of urination, and moderate cystitis of the vesical neck.

This was a year ago. He recovered under six weeks of methodic treatment without further accident, and remains well.

After this, upon reading the paper by Dr. Curtis already alluded to, I determined to test the method of deep urethral irrigation followed by an astringent in a few cases personally.

My experience covers five cases, in the management of which I was assisted by Dr. Blackwell.

CASE I.—A gentleman, an old offender, with a tough urethra, came to me within a few hours of the commencement of his urethral discharge, which was very mild.

His urethra was irrigated and the tannin solution applied according to the method advocated by Dr. Curtis. Twenty-four hours later he returned in considerable pain with swollen meatus, a faint watery discharge, pain on urinating, and considerable inflammation and œdema of the entire foreskin and a portion of the integument covering of the penis. He declined further injection. His penis was wrapped up in a hot lead-and-opium solution and an alkaline diuretic with an anodyne was ordered. The swelling promptly declined and in a few days he became and remained well without further local treatment. He had been drinking freely before this attack, and I therefore concluded that he had not had a gonorrhœa, but an irritation of some of his old tender urethral spots.

However, both the patient and myself were inclined to think well of the method since the ultimate result had been good.

As it so happened, some weeks later, again after drinking and suspicious sexual contact, he once more appeared with slight pain on urination and a distinct urethral discharge. I urged him again to submit to the irrigation, but he declined on the score of some pressing duties and asked for a mild injection to be applied in the usual way, stating that after a few days, if the discharge continued, he would have performed his pressing duties and would then again submit to the hot irrigation.

I ordered a mild sulpho-carbolate of zinc injection and he improved from the very first application, so that after a few days he was so manifestly on the high-road to recovery that he declined to allow the hot irrigation to be used. He recovered entirely in a few days, thus justifying his wisdom in refusing the harsher method, and proving to my satisfaction that neither of his attacks had been truly gonorrhœal.

CASE II.—A gentleman, past middle life, was treated by the Curtis method for his first gonorrhœal attack. No inflammation of the urethral or surrounding structures followed. The injections were repeated a number of times during ten days. Each one was followed by a temporary subsidence of the discharge, which promptly recurred. At first the injections were repeated daily, then at longer intervals. After ten days they were stopped and ordinary treatment instituted, under which he slowly recovered, the entire time being about five weeks.

CASE III.—The first injection in this patient, who was a young man with a pale face but in good health, and suffering from true gonorrhœa, caused the penis to swell along its entire length, increased the pain and (after twenty-four hours) the discharge to such an extent that he refused further trial of the method and went on with an aggravated gonorrhœal attack lasting about three months, and more or less complicated by mild gonorrhœal cystitis.

CASE IV.—Was a fresh case of true gonorrhœa in a mulatto. But little swelling followed the irrigations. The discharge was temporarily arrested by each injection, and then went on as before. After three weeks the method was given up, and a slow cure effected by ordinary means.

CASE V. was a counterpart of Case II.

In these five cases the utmost care was used in making the injections, which were done without any violence, a very small rubber soft catheter being used and a fountain syringe.

My experience comprises two other cases treated primarily by other physicians.

CASE I.—During the summer of 1883 I was called in consultation by a gentleman having charge of a case which had been under the care of still another medical man. I was informed that the patient had been submitted to the deep urethral hot-water irrigation method for the cure of a gonorrhœa.

When I saw the patient he was confined to his room in bed with a high fever and great perineal pain. His discharge was better than it had been, but his prostate by the rectal touch was found to be very seriously congested, hot, tense, throbbing, and all the indications pointed toward probable prostatic abscess.

By methodical treatment he slowly improved, there was no abscess, the urethral discharge came back—as is its wont—when the prostatic swelling subsided. When I last heard from his physician, recovery was assured. I cannot state the exact number of weeks during which this patient's discharge lasted.

CASE II.—This case is the most brilliant of all. The patient is now on his back in bed at the end of the eighth week with a free urethral discharge.

The history is as follows: In July, 1883, one year ago, this patient came to me with his first attack of gonorrhœa.

I knew that the family was (urethrally speaking) an inflammable one. The patient had only three brothers. One had twice been under my care with bad attacks of gonorrhœal rheumatism. The second, with a prolonged gonorrhœa of many months' duration, treated in the country, had had an inflamed inguinal gland which required many months for its dissipation without suppuration. The third had a sharp urethral discharge which lasted him the better part of a year.

With such knowledge I treated the patient most carefully, avoiding injections. He recovered in about two months, with no complication greater than a little urgency upon urinating toward the close of his treatment.

Eight weeks ago, while sitting in Delmonico's, he became conscious of a slight urethral discharge. He confided the fact to a friend sitting beside him; and his horror of the disease which had been two months in getting well a year before. The friend said to him that there was no need of being so long as that getting well of gonorrhœa, that he would take him to a doctor who would "fix him in a week." True prophet, alas! for in a week the patient certainly was "fixed"—upon his bed, where he has remained ever since. The doctor, be it understood, was a thoroughly competent practitioner.

The method was as follows: Every hour while awake during the day the patient was told to inject his urethra—with an ordinary syringe—with water as hot as the urethra could tolerate, and three times a day he was to take a full hot bath, and while in the bath to inject his urethra under water with the hot water of the bath *as many times as possible.* This he faithfully did for a week. On the fourth day he began to feel pain on urination deep in the perineum. His calls to urinate became frequent and urgent. The doctor said that this was not important, and ordered him over and above the hot water to use an astringent injection. This the patient did, and, his sufferings steadily aggravating, he sent for me on the eighth day. He said that the treatment had certainly been effective in stopping the discharge, but he had the piles frightfully, and the doctor had given him an ointment to apply outside. He was, however, growing suspicious of the treatment, so he sent word to the doctor that he was well and would call upon him shortly, and then hurried a messenger to me. Possibly he may yet come to be recorded by his physician as a cure of gonorrhœa by hot water injections in a week.

I found the temperature 104°, intense perineal pain and urgency of urination, no pus flowing from the urethra, but plenty of it in the urine—in short, the case was one of gonorrhœal cystitis and prostatitis of a high grade, induced by the peculiar treatment I have detailed above.

The cystitis to-day—seven weeks later—is well, the prostatitis nearly so. There has been no abscess, but the patient has had active epididymitis, which has relapsed three times, the patient being all the while kept flat upon his back in bed. A new wave of inflammation, without known cause, would seem to pass over his prostatic sinus and vesical neck, and shortly there would be a relapse of the inflammation in the testicle. Now the testicle is reasonably quiet, and the urethral discharge has, naturally, returned in creamy abundance. Dr. Samuel Alexander two days ago found an abundance of gonococci in the urethral pus.

As for other methods of aborting gonorrhœa. I have not tried eucalyptol, but I have experimented with iodoform suppositories, and with frequent injections of weak corrosive sublimate solutions.

The iodoform bougies have failed me totally, but have done no harm. I have used those of Kelly and Durkee, two grains, coumarin one-twentieth grain each.

With corrosive sublimate injections I commenced with one-quarter grain to the ounce of water, but found it too strong for frequent use in a virgin case. Some old stagers liked it and confessed moderately good results as having followed its use in their cases of spurious gonorrhœa. I then reduced to one sixth grain in the ounce, but was totally disappointed in its use. With such a solution injected three or four times a day, I did not succeed in aborting a single case of gonorrhœa out of several in which it was tried.

On one occasion, after a plastic operation upon a healthy urethra to close a fistula in the scrotal portion, I attempted to irrigate the urethra by a small rubber catheter passed beyond the point of the former fistula with a one-in-two-thousand (one-quarter grain to the ounce) solution of corrosive sublimate, a solution with which I had washed the wound thoroughly and with which surgeons freely irrigate ordinary wounds without causing irritation.

The soft-rubber catheter could only be passed once beyond the scrotal point where the fistula had been. The urethra swelled so much that it became temporarily occluded at that point, and after two days an abundant creamy suppuration occurred under a continuance of the injections which persisted for a number of days after the injections had been suspended.

My conclusions, therefore, are—my temporary conclusions, I should say, for they are based on too imperfect data to allow accurate generalization—

1st. A mild bichloride of mercury solution irritates the mucous membrane of the urethra more than it seems to irritate an open wound.

2d. It appears that an abortive treatment of true gonorrhœa is yet to be discovered.

3d. The hot-water treatment of gonorrhœa is unreliable.

